# Efficacy and moderators of cognitive therapy versus behavioural activation for adults with depression: study protocol of a systematic review and meta-analysis of individual participant data

**DOI:** 10.1192/bjo.2022.560

**Published:** 2022-08-10

**Authors:** Ellen Driessen, Zachary D. Cohen, Lorenzo Lorenzo-Luaces, Steven D. Hollon, David A. Richards, Keith S. Dobson, Sona Dimidjian, Jaime Delgadillo, Fernando L. Vázquez, Kathleen McNamara, John J. Horan, Pauline Gardner, Tian P. Oei, Anuj H. P. Mehta, Jos W. R. Twisk, Ioana A. Cristea, Pim Cuijpers

**Affiliations:** Department of Clinical Psychology, Behavioural Science Institute, Radboud University, The Netherlands; and Depression Expertise Center, Pro Persona Mental Health Care, The Netherlands; Department of Psychiatry and Biobehavioral Sciences, University of California, Los Angeles, USA; Department of Psychological and Brain Sciences, Indiana University Bloomington, USA; Department of Psychology, Vanderbilt University, USA; Department of Health and Community Science, Faculty of Health and Life Sciences, University of Exeter, UK; and Department of Health and Caring Sciences, Western Norway University of Applied Sciences, Norway; Department of Psychology, University of Calgary, Canada; Crown Institute and Department of Psychology and Neuroscience, University of Colorado Boulder, USA; Clinical and Applied Psychology Unit, University of Sheffield, UK; Department of Clinical Psychology and Psychobiology, University of Santiago de Compostela, Spain; Private Practice, Colorado, USA; Counseling Psychology Program, Arizona State University, USA; Caulfield Pain Management and Research Centre, Caulfield Hospital, Australia; School of Psychology, The University of Queensland, Australia; Department of Psychology, University of Toronto Scarborough, Canada; Department of Epidemiology and Biostatistics, Amsterdam University Medical Centers, The Netherlands; Department of Brain and Behavioral Sciences, University of Pavia; and IRCCS Mondino Foundation, Italy; Department of Clinical, Neuro and Developmental Psychology, Amsterdam Public Health Research Institute, Vrije Universiteit Amsterdam, The Netherlands

**Keywords:** Depressive disorders, cognitive–behavioural therapies, behavioural activation, individual participant data meta-analysis, moderators

## Abstract

**Background:**

Cognitive therapy and behavioural activation are both widely applied and effective psychotherapies for depression, but it is unclear which works best for whom. Individual participant data (IPD) meta-analysis allows for examining moderators at the participant level and can provide more precise effect estimates than conventional meta-analysis, which is based on study-level data.

**Aims:**

This article describes the protocol for a systematic review and IPD meta-analysis that aims to compare the efficacy of cognitive therapy and behavioural activation for adults with depression, and to explore moderators of treatment effect. (PROSPERO: CRD42022341602)

**Method:**

Systematic literature searches will be conducted in PubMed, PsycINFO, EMBASE and the Cochrane Library, to identify randomised clinical trials comparing cognitive therapy and behavioural activation for adult acute-phase depression. Investigators of these trials will be invited to share their participant-level data. One-stage IPD meta-analyses will be conducted with mixed-effects models to assess treatment effects and to examine various available demographic, clinical and psychological participant characteristics as potential moderators. The primary outcome measure will be depressive symptom level at treatment completion. Secondary outcomes will include post-treatment anxiety, interpersonal functioning and quality of life, as well as follow-up outcomes.

**Conclusions:**

To the best of our knowledge, this will be the first IPD meta-analysis concerning cognitive therapy versus behavioural activation for adult depression. This study has the potential to enhance our knowledge of depression treatment by using state-of-the-art statistical techniques to compare the efficacy of two widely used psychotherapies, and by shedding more light on which of these treatments might work best for whom.

According to the World Health Organization, depression is the single largest contributor to global disability.^1^ It affects more than 300 million people around the world and is a major contributor to the death by suicide of 800 000 people each year.^1^ Given the tremendous burden that depression poses on individuals and on society as a whole, there is a pressing need for effective depression treatments. Many people with depression prefer to be treated with psychotherapy rather than antidepressant medication.^2^ Cognitive therapy and behavioural activation are both efficacious psychotherapies for depression^3^ that are recommended as first-line interventions in depression treatment guidelines.^4,5^

Cognitive therapy is based on the cognitive model of depression, which posits that biased beliefs and maladaptive information processing have a causal role in the development and maintenance of depression. Cognitive therapy, therefore, aims to correct maladaptive thinking and beliefs to reduce acute distress and the risk for subsequent symptom return.^6^ Behavioural activation, on the other hand, is based on behavioural principles and the premise that depressive behaviour can be best understood in context and as a function of reinforcement contingencies.^7,8^ Although early behavioural therapies for depression mainly aimed to increase a person's access to positively reinforcing stimuli,^7^ newer forms also focus on reducing avoidance, increasing mindfulness and providing a functional-analytic understanding of the relationship between behaviour and mood.^8^ A core technique in behavioural activation is activity scheduling, whereby individuals monitor their mood and daily activities to learn the connection between them, and then focus on increasing activities that are expected to result in a sense of pleasure, mastery or accomplishment.^7^ This behavioural intervention is also part of cognitive therapy for depression^6^ and the term cognitive–behavioural therapy is often used in the literature to denote a single depression intervention that includes both a behavioural activation and a cognitive restructuring component. In addition to activity scheduling, cognitive therapy and behavioural activation overlap in being present-focused, symptom-focused, structured, directive and time-limited.

## Comparative efficacy of cognitive therapy and behavioural activation

Interest in behavioural activation increased considerably after a dismantling study suggested that the behavioural activation component of cognitive therapy alone performed as well as the full cognitive therapy package (behavioural activation plus modifying automatic thoughts and core depressogenic schemata), challenging the notion that maladaptive thinking needs to be directly addressed to reduce depression.^9^ Despite its influence in the field, this dismantling study was underpowered to detect small treatment effects. Similar findings, however, were later obtained in a large and adequately powered trial showing non-inferiority of behavioural activation to cognitive therapy in the treatment of depression.^10^ In fact, a recent study of internet-based cognitive–behavioural therapies (iCBTs) suggested that the behavioural activation component contributed to the efficacy of iCBT, but the cognitive therapy component did not;^11^ however, it is unclear whether these findings extend to comparisons of face-to-face cognitive therapy and behavioural activation. Because behavioural activation is a simpler intervention than cognitive therapy and requires less intensive and costly training, it is currently receiving increased research attention as a potentially cost-effective depression treatment.^10^

Conventional meta-analyses comparing the efficacy of cognitive therapy and behavioural activation have reported no significant differences on post-treatment and follow-up measures of depression,^12,13^ or with regard to treatment acceptability, quality of life, anxiety symptoms and social functioning.^13^ A recent network meta-analysis also found no evidence for differences in effectiveness between cognitive restructuring, cognitive–behavioural therapy and behavioural activation.^14^ These findings suggest that behavioural activation might indeed be as efficacious as cognitive therapy in the treatment of depression. However, these meta-analyses are based on study-level data extracted from publications, and therefore depend on the quality of the published information. Because treatment effects can be overestimated in published trial reports,^15^ such meta-analyses can potentially lead to biased results.^16^

## What works for whom?

It is largely unclear which individuals might benefit more from cognitive therapy compared with behavioural activation, and vice versa. One study reported that behavioural activation was more efficacious than cognitive therapy for participants with more severe depression.^17^ However, this finding was *post hoc* and was not replicated in two other studies.^10,18^ Also, a psychotherapy process study suggested that participants with more severe depression benefitted less from behavioural interventions compared with participants with less severe depression, although cognitive techniques predicted outcomes regardless of severity.^19^ Thus, the evidence for baseline depression severity moderating cognitive therapy versus behavioural activation efficacy is inconclusive. Additionally, a recent pilot study found that cognitive skills and behavioural avoidance were related to differential efficacy of cognitive therapy and behavioural activation, but these findings have yet to be replicated.^20^

One of the reasons why it is largely unclear which of these treatments works best for whom is the lack of statistical power in individual clinical trials.^21^ These are typically powered to identify an intervention effect, but to examine which participants will respond best to which treatment in a randomised trial, much larger sample sizes are needed.^22^ Conventional meta-analyses^23^ are also not well-suited to study moderators because of limited statistical power and because they are prone to ecological fallacy, such that the association between the study-level characteristics may not be representative of the true relationships in the data at the individual level.^24^ Thus, both individual clinical trials and conventional meta-analyses are insufficiently able to answer the question as to whether individuals with certain characteristics benefit more from cognitive therapy or behavioural activation.

## Individual participant data meta-analysis

An alternative method to examine treatment effects is individual participant data (IPD) meta-analysis.^25^ IPD meta-analysis combines participant-level data from multiple clinical trials, which increases statistical power relative to both individual clinical trials and conventional meta-analysis. Furthermore, the IPD technique has several additional advantages over the latter. First, by applying the same analytic approaches for handling missing data and statistical modelling, IPD meta-analysis facilitates standardisation across studies. Second, it can verify the original studies’ results and use novel statistical methods that were not yet available at time of publication. Third, IPD is particularly suited to examine moderators of treatment efficacy, not only because of the increased statistical power, but also because ecological fallacy can be circumvented when working with data at the participant level.^26^ As such, IPD meta-analyses can provide important information on participant characteristics that might be related to differential treatment response; however, their findings are observational and require validation before they can be used to guide treatment selection.

This study, therefore, will be a systematic review and IPD meta-analysis that aims to (a) compare the efficacy of cognitive therapy and behavioural activation as assessed on a range of outcomes at post-treatment and follow-up in randomised clinical trials for adults with depression, and (b) examine the potential moderating effects of baseline depression severity and other participant characteristics on post-treatment depressive symptom measures. This article describes the protocol for this study.

## Method

### Protocol and registration

This study will build on and extend prior work by our group.^27–30^ It is registered in the PROSPERO International Prospective Register of Systematic Reviews (PROSPERO registration number: CRD42022341602). Additional important protocol amendments will also be documented in the PROSPERO register. This documentation will include the date of the amendment, a description of the change and the rationale. This article is reported in accordance with the Preferred Reporting Items for Systematic Review and Meta-Analysis Protocols (PRISMA-P) statement.^31^

### Eligibility criteria

This meta-analysis will include (a) randomised clinical trials (b) comparing cognitive therapy and behavioural activation (c) for the acute-phase treatment of depression (d) in adults. No restrictions will be placed on the years when the study was conducted, publication language, publication date or publication status.

Studies will be included if they directly compare cognitive therapy and behavioural activation among participants who were randomly assigned to these treatments. An intervention will be considered to be cognitive therapy if it is a manualised psychotherapy with cognitive restructuring as the main treatment component.^27^ Behavioural techniques (including activity scheduling) will be allowed, as long as they are part of an intervention protocol that is aimed at cognitive restructuring. Beck et al's model^6^ is considered the cognitive therapy prototype, but other models can also be included. An intervention will be considered to be behavioural activation if the core element of treatment is aimed at increasing positive reinforcement by means of activity scheduling. Inclusion of cognitive restructuring techniques will not be allowed. As such, behavioural activation encompasses the early intervention developed by Lewinsohn,^7^ which mostly involves mood monitoring and scheduling pleasant activities, as well as newer interventions, such as the one developed by Jacobson et al,^8^ which additionally includes functional analysis and a focus on reducing avoidance behaviours, and the behavioural activation approach studied extensively by Lejuez et al.^32^ Any cognitive therapy and behavioural activation format will be allowed (e.g. individual, group), as will any delivery method (e.g. telephone, e-mail, videoconferencing) as long as a care professional delivers the therapy. Thus, unguided bibliotherapy or unguided internet interventions will be excluded. Guided bibliotherapy, guided internet therapy or other guided self-help formats will be included if provided by a trained healthcare worker. No restrictions will be placed on the setting where the psychotherapies are conducted, the number of sessions or the duration of follow-up.

Following previous depression treatment IPD meta-analyses,^29,30^ participants will be considered to have depression if they meet specified criteria for a unipolar mood disorder assessed by means of a semi-structured interview or clinician's assessment, or if they present a score above the validated cut-off on an evaluator-assessed, clinician-assessed or self-reported measure of depression. Comorbid mental and somatic disorders will be allowed to increase generalisability of this study's findings to clinical practice. Thus, studies allowing comorbidities or focusing specifically on comorbidities (e.g. depression and substance misuse) will be included. As this study assesses the acute-phase treatment of depression, studies in which cognitive therapy and behavioural activation are examined as maintenance or relapse prevention treatments after the acute treatment phase will be excluded.

Participants must be at least 18 years old. Thus, studies for child or adolescent depression will be excluded, but studies including older adult populations will be considered. Participant criteria will be assessed at study level. In line with prior depression treatment IPD meta-analyses,^29,30^ eligible participants from a study including a wider population (e.g. adults from a study including both adolescents and adults) will not be included, because the integrity of randomisation within the subgroup of eligible participants could be compromised.

### Information sources, search strategy and selection process

A database of randomised clinical trials examining the efficacy of psychological treatments for depression will be searched to identify relevant studies (www.metapsy.org). This METAPSY database has been used in a series of published meta-analyses and is updated annually. It is developed through comprehensive literature searches in the bibliographic databases PubMed, PsycINFO, EMBASE and the Cochrane Library. The search strings use a combination of index terms and free-text words indicative of depression and psychotherapies, with filters for randomised clinical trials. The exact terms for the searches are available from https://osf.io/nv3ea/.

Two raters will independently screen all records based on titles, abstracts and keywords, will assess full-text papers for METAPSY database eligibility and will rate the treatment comparison(s) examined. Disagreements will be resolved through consensus. Next, two raters will independently assess all full-text papers of studies marked as comparing a psychotherapy monotreatment condition against another active monotreatment condition, for meeting the eligibility criteria for this work. Disagreements will again be resolved through consensus. In addition, prior behavioural activation for depression meta-analyses^12–14^ and reference lists of the included studies will be checked for studies meeting the eligibility criteria that might have been missed.

### Data collection process

Authors of the included studies will be invited to participate in this project by using a strategy that has been successful in soliciting participation in previous depression treatment IPD meta-analyses.^27–30^ Authors sharing their IPD will be offered co-authorship for all publications resulting from the use of these data, inasmuch as they meet internationally accepted criteria for authoring scientific articles (www.icmje.org). In addition, the aggregated database will be made available to investigators who share IPD to examine other research questions, provided that the authors of the original studies approve the use of their data for this purpose.

To invite authors, a multi-step contact protocol will be applied, which has previously been described^30^ and has proven to be successful in reaching authors for prior depression treatment IPD meta-analyses. More specifically, contact details of all corresponding authors will be collected from the relevant publications, through internet searches or personal contacts with other researchers. Corresponding authors will be sent an email invitation outlining the project's goals and asking for their collaboration by sharing their study's IPD. If the corresponding author does not respond after 3 weeks, a second and third email will be sent. If no response is received from the corresponding author, the other authors will be contacted in the same way, in this order: first, last, second, third, fourth, etc. If none of the authors respond to the emails, a letter will be mailed to the corresponding author (again with three attempts). If no response to these letters is received, the corresponding author will be contacted by telephone. If the corresponding author does not respond, the other authors will be contacted by letter and telephone. If none of the authors responds to these efforts, other ways will be sought to contact one of the authors (e.g. via colleagues who might know them). A study's data will be considered unavailable only if (a) all of these attempts fail or (b) an author either indicates that the IPD were not retained or declines to share these data.^29,30^

### Data items

The following participant-level data items will be requested: treatment condition, all outcome variables assessed during and after treatment (with item-level data for depression outcome measures) and all potential moderator variables assessed in the study. Moderator variables are defined as all demographic (e.g. age, gender), clinical (e.g. depressive episode duration, comorbid anxiety disorder) and psychological (e.g. personality, coping style) participant characteristics assessed in the study before the start of treatment. The primary study's authors will anonymise the participant-level data-set before transferring it.

The following study-level characteristics will be extracted from the publications: country where the study took place, recruitment method (e.g. community, clinical), target group (e.g. adults in general, students), depression inclusion criteria, number of cognitive therapy/behavioural activation sessions, cognitive therapy/behavioural activation format (e.g. individual, group) and assessment time points. Cognitive therapy and behavioural activation treatment quality will be examined with regard to use of a treatment manual, provision of therapy by trained therapists and verification of treatment integrity. In addition, effect size data will be extracted from the published articles. If information on study-level characteristics or treatment quality, or effect size data, is not reported in the publications, it will be requested from the authors.

### Data integrity checks

Upon receiving the data-set, three data integrity checks will be performed. First, it will be checked whether the data-set includes the full intention-to-treat sample (i.e. all participants randomised to treatment) and otherwise matches the data reported in the published article. To this end, all baseline characteristics, and observed mean pre-treatment and post-treatment scores reported in the article, will also be calculated from the data-set and both will be compared. Second, it will be checked whether all outcome and potential moderator variables reported in the article are included in the data-set. Third, the outcome and moderator variables will be checked for inconsistent, invalid or out-of-range items. Discrepancies resulting from these data integrity checks will be resolved with the authors.

After the integrity checks have been performed, the data-sets will be standardised and merged in the IPD meta-analysis database. For this purpose, relevant variables will be extracted from each trial's raw data file, recoded and copied into a single database in which each participant is identified by a study identifier and a unique individual participant identifier. After all data files have been recoded and entered, the data for each study will be checked with the original data file for accuracy.^29,30^

### Outcomes and prioritisation

Depressive symptom level at treatment completion will be the primary outcome of this study, as symptom reduction is considered to be the main aim of cognitive therapy and behavioural activation in the acute-phase treatment of depression. Depressive symptom level at treatment completion is operationalised as a participant's score on the primary continuous depression scale administrated at the primary post-treatment time point, both as defined by the study's authors. Continuous rather than dichotomous measures (e.g. remission) were chosen as primary outcome because of the increased variance that might facilitate moderator identification. Secondary outcomes will be depressive symptom level at follow-up, as well as all other outcomes at post-treatment or follow-up that are assessed in at least two studies (e.g. anxiety symptoms, interpersonal functioning, quality of life).

Individual studies are expected to use different instruments to assess depressive symptoms as well as the secondary outcome domains. Outcomes will therefore be standardised by converting raw scores into *z*-scores within each study.^29,30^ Sensitivity analyses will be conducted with unstandardised scores for each specific depression measure that is assessed in the majority of studies included in the meta-analysis.^29,30^ The meta-analyses’ principal measures of effect will be Cohen's *d* effect sizes for analyses with *z*-scores as outcome measure, and mean differences for analyses with unstandardised scores as outcome measure. Cohen's *d* effect sizes of 0–0.32, 0.33–0.55 and 0.56–1.2 will be considered small, moderate and large, respectively.^33^

Potential moderators will include all demographic, clinical and psychological participant characteristics that are assessed in at least two studies. These are likely assessed differently in individual studies and will be standardised as well; for instance, by converting scores into *z*-scores for continuous variables or by recoding variables into similar categories for categorical variables.

### Risk of bias in individual studies

Risk of bias in the included studies will be assessed with the Cochrane risk-of-bias tool for randomised trials.^34^ Two raters will independently assess this tool at outcome level. Disagreements will be resolved by consensus. Ratings will be primarily based on information reported in the publications, although selection bias and reporting bias will be assessed with the IPD. As studies are expected to be included that were published before the universal adoption of reporting guidelines for randomised clinical trials,^35^ requisite information will be requested from the authors if it is not reported in the publications.

### Data synthesis

To facilitate the comparison of this study's findings to prior work, a data-analysis strategy will be adopted that has been previously applied in depression treatment efficacy IPD meta-analyses, and is described in more detail elsewhere.^30,36^ This strategy is based on the approach recommended by Twisk et al,^37^ and will be used because it adequately accounts for baseline values and has favourable properties concerning missing data (allowing participants with only a baseline value but missing post-treatment and/or follow-up assessments to remain included in the analyses).

One-stage IPD meta-analyses will be conducted in intention-to-treat samples, using mixed-effects models with restricted maximum likelihood estimation. Because additional help-seeking usually cannot be controlled during the follow-up period, follow-up data will be excluded from post-treatment analyses. Heterogeneity will be assessed by calculating the variance between studies as a proportion of the total variance (i.e. *I^2^*-statistic). The normality assumption will be checked by inspecting histograms of residuals.

To compare the efficacy of cognitive therapy and behavioural activation, a model will be estimated including a main effect for time (categorical; represented by dummy variables) and a time×treatment interaction,^37^ a random intercept with respect to study (to account for the clustering of participants within studies) and a random intercept with respect to participants (to account for the clustering of repeated measures within participants). The starting model contains fixed slopes, but the –2 log likelihood change will be evaluated to decide whether to add a random slope for the time×treatment interaction at study level. The regression coefficient of the time×treatment interaction will be considered to indicate the magnitude of the treatment effect.

To examine moderator effects, first, a model will be estimated for each potential moderator variable. This model will comprise the model described above, with an additional main effect for the moderator and a time×moderator×treatment three-way interaction. To deal with the issue of multiple analyses, all potential moderators with a *P*-value <0.10 for the three-way interaction's regression coefficient will next be included in a model simultaneously. A significant (*P* < 0.05) regression coefficient of the three-way interaction in this final model will be considered to indicate a moderating effect. For categorical moderator variables, separate treatment effects will be estimated for each moderator category if a significant moderating effect is found.

Because sample sizes can vary between variables depending on the data available, effect sizes for each outcome and moderator will also be examined independently of their *P*-values, to consider whether type 1 or type 2 errors might have been made. For each outcome and potential moderator, the strength of the body of evidence will be assessed based on the number of included studies and participants, as well as the quality of the included studies.

### Sensitivity analyses

Six sensitivity analyses will be conducted with regard to the primary efficacy analysis. First, as described previously, the analysis will be repeated with unstandardised scores for each specific depression measure that is assessed in the majority of studies included in the meta-analysis.^29,30^ Second, to examine the robustness of the findings to those eligible for cognitive therapy and behavioural activation for depression in general mental healthcare, the analysis will be repeated in studies including general adult populations meeting diagnostic criteria for a mood disorder and providing face-to-face cognitive therapy and behavioural activation. Third, to examine the impact of risk of bias in the primary studies, risk-of-bias items will be added as dichotomous covariates to the mixed-effects model. Fourth, the impact of cognitive therapy and behavioural activation quality will be examined in the same way. Fifth, to examine potential differences in treatment effects, the analysis will be conducted separately in the subgroup of studies excluding behavioural techniques in cognitive therapy and the subgroup of studies including behavioural techniques in cognitive therapy. Sixth, to differentiate between Beckian cognitive therapy and other approaches, the analysis will be conducted separately in the subgroup of studies that cite the Beck treatment manual for cognitive therapy^6^ and the subgroup of studies that refer to other treatment manuals.

In addition, one sensitivity analysis will be conducted with regard to the moderation analyses. To examine the possibility that false conclusions are drawn about what might be important moderators today based on data collected at a time in the past, when some of these factors may have had a different level of importance, we will repeat the moderator analyses excluding studies that were completed in the 1970s and 1980s.

### Meta-biases

Following the recommendations proposed by Sterne et al,^38^ potential publication bias will be assessed by examining asymmetry in contour-enhanced funnel plots with Egger's test for analyses including ten or more trials. Potential data availability bias will be assessed by comparing characteristics between studies for which IPD were and were not obtained with *t*-tests for continuous variables and *χ*^2^-test analyses for categorical variables. In addition, Cohen's *d* effect sizes will be calculated based on the effect size data extracted from the publications. Conventional meta-analysis ‘subgroup analyses’ will then be conducted – applying a fully random-effects analysis and pooling study-to-study variance across subgroups – to compare effect sizes between studies for which IPD were and were not available.

### Ethics statement

This study did not require institutional review board (IRB) approval. Depending on their institution's policies, IRB approval may be required for the authors to share their IPD. If their institution's policies require them to do so, it is the authors’ responsibility to obtain IRB approval. With signing the data-sharing agreement, investigators sharing IPD declare that these IPD were collected and are transferred to our research group in accordance with all applicable local and international laws and regulations. Furthermore, they declare that all IPD will be anonymised, so that no personal data are transferred.

## Discussion

This article described the protocol for a systematic review and IPD meta-analysis examining the efficacy and moderators of cognitive therapy versus behavioural activation for adult depression. The goal of this study is to identify randomised clinical trials comparing these two treatments by means of a systematic literature search, and to collect their IPD. Additionally, the goal is to compare the efficacy of cognitive therapy and behavioural activation as assessed on a range of outcomes at post-treatment and follow-up, and to examine the potential moderating effects of various demographic, clinical and psychological participant characteristics on post-treatment measures of depressive symptoms.

### Strengths and limitations

This study has a number of strengths related to its IPD meta-analytic design. First, standardisation across the primary studies is facilitated by using the same statistical approach to estimate treatment and moderator effects. Moreover, analyses will be based on intention-to-treat samples, adequately correcting for baseline values in all studies. This is expected to result in more precise effect estimates relative to previous conventional meta-analyses. Second, requesting all outcome and all potential moderator variables assessed in the studies increases the chances of accessing data that might not have been reported in publications. Third, and most importantly, IPD meta-analytic methods allow for examining moderators of treatment effect at the individual participant level with increased statistical power.^39^

This study also has a number of limitations related to its IPD meta-analytic design. First, collecting, checking, processing and analysing IPD is a time-consuming and labour-intensive process. It therefore takes more time and resources to conduct an IPD compared with a conventional meta-analysis. A governmental research grant provides the necessary financial support for this study, and the investment is offset by the increased quality of the analyses and reliability of the results. Second, IPD meta-analyses rely on data collected in trials that have already been completed. Thus, it is possible that not all outcome or potential moderator variables of interest can be examined. Meta-analyses of different outcomes or moderators might also be based on different subgroups of studies. Relatedly, constructs will likely be operationalised differently between studies, making it necessary to standardise variables to conduct meta-analyses including all relevant studies. To recode categorical variables into similar categories, the largest common denominator across studies will need to be used, which can result in loss of information for some studies. Third, both cognitive therapy and behavioural activation originated in the 1970s and have been studied since the early days of their development. As such, some trials comparing their efficacy might have been completed more than 40 years ago, and it is likely that IPD of these studies might not be available. However, as early psychotherapy trials often had very small samples (*n* = 8–10 per treatment condition), it is expected that this will not substantially affect this study's total sample size. Data availability bias will be assessed empirically. Fourth, the findings of this study will be observational. Significant moderator effects will thus require validation before they can be used to guide treatment selection.

### Clinical and scientific relevance

Cognitive therapy and behavioural activation are widely used psychotherapies for depression, and both are included as therapeutic options in depression treatment guidelines.^4,5^ Cognitive therapy is considered by many to be the ‘gold-standard’ psychotherapy for depression,^40^ whereas behavioural activation has received increased attention in recent years because of its potential of being a cost-efficient alternative to cognitive therapy. For this reason, it is of considerable clinical importance to assess the relative efficacy of cognitive therapy and behavioural activation in the treatment of depression. An IPD meta-analysis can add to the available body of conventional meta-analytic evidence by providing more precise effect estimates. To the best of our knowledge, this will be the first IPD meta-analysis concerning the efficacy of cognitive therapy versus behavioural activation for adult depression.

Moreover, it is largely unclear which individuals might benefit more from one of these treatments than the other. Baseline depression severity may be a particularly promising effect modifier. IPD meta-analysis can add to the available literature in this regard by studying moderators at the individual participant level with increased statistical power. To the best of our knowledge, this will also be the first study examining moderators across clinical trials that assess the efficacy of cognitive therapy and behavioural activation. Thus, this study has the potential to enhance our knowledge of depression treatment by using state-of-the-art statistical techniques to compare the efficacy of two widely used psychotherapies, and by shedding more light on which of these treatments works best for whom.

## Data Availability

Data availability is not applicable to this article as no new data were created or analysed for this study protocol paper.
